# Complete Chloroplast Genome of the Wollemi Pine (*Wollemia nobilis*): Structure and Evolution

**DOI:** 10.1371/journal.pone.0128126

**Published:** 2015-06-10

**Authors:** Jia-Yee S. Yap, Thore Rohner, Abigail Greenfield, Marlien Van Der Merwe, Hannah McPherson, Wendy Glenn, Geoff Kornfeld, Elessa Marendy, Annie Y. H. Pan, Alan Wilton, Marc R. Wilkins, Maurizio Rossetto, Sven K. Delaney

**Affiliations:** 1 National Herbarium of New South Wales, Mrs Macquaries Road, Sydney, NSW, 2000, Australia; 2 School of Biotechnology and Biomolecular Biosciences, University of New South Wales, Kensington, NSW, 2033, Australia; 3 Hanze University of Applied Sciences, Groningen, Zernikeplein 7, 9747, AS Groningen, The Netherlands; Universidad Miguel Hernández de Elche, SPAIN

## Abstract

The Wollemi pine (*Wollemia nobilis*) is a rare Southern conifer with striking morphological similarity to fossil pines. A small population of *W*. *nobilis* was discovered in 1994 in a remote canyon system in the Wollemi National Park (near Sydney, Australia). This population contains fewer than 100 individuals and is critically endangered. Previous genetic studies of the Wollemi pine have investigated its evolutionary relationship with other pines in the family Araucariaceae, and have suggested that the Wollemi pine genome contains little or no variation. However, these studies were performed prior to the widespread use of genome sequencing, and their conclusions were based on a limited fraction of the Wollemi pine genome. In this study, we address this problem by determining the entire sequence of the *W*. *nobilis* chloroplast genome. A detailed analysis of the structure of the genome is presented, and the evolution of the genome is inferred by comparison with the chloroplast sequences of other members of the Araucariaceae and the related family Podocarpaceae. Pairwise alignments of whole genome sequences, and the presence of unique pseudogenes, gene duplications and insertions in *W*. *nobilis* and Araucariaceae, indicate that the *W*. *nobilis* chloroplast genome is most similar to that of its sister taxon *Agathis*. However, the *W*. *nobilis* genome contains an unusually high number of repetitive sequences, and these could be used in future studies to investigate and conserve any remnant genetic diversity in the Wollemi pine.

## Introduction

The monotypic gymnosperm *Wollemia nobilis* W.G. Jones, K.D. Hill & J. M. Allen (or Wollemi pine) was discovered in 1994 in the secluded warm temperate rainforests of the Wollemi National Park, New South Wales, Australia [1]. *W*. *nobilis* is similar to fossil pines from the Cretaceous period (approximately 140 million years ago) and relatives of *Wollemia* were once widespread [2,3], but the living population consists of fewer than 100 individuals confined to a single canyon system. This critically endangered species belongs to the Araucariaceae, a conifer family containing 30 species and three extant genera (*Agathis*, *Araucaria*, *Wollemia)* [4–7]. The current distributions of Araucariaceae and the closely related family Podocarpaceae are predominantly in the Southern Hemisphere [8,9]. *W*. *nobilis* can reach up to 40 m in height [1] and has the ability to form new vertical branches through coppicing [10]. Coppicing can occur in *Agathis* and *Araucaria* in response to trauma, but only *Wollemia* grows regularly in this manner.

Morphology alone does not resolve the position of *Wollemia* within the Araucariaceae [1,11], but phylogenetic studies using several chloroplast genes and ribosomal DNA data have placed *Wollemia* as sister to *Agathis* [4,5,7,12]. Molecular dating suggests that *Wollemia* and *Agathis* last shared a common ancestor between 55 and 90 million years ago [13,14]. This broad range reflects several incongruities within the literature regarding fossil calibrations and affinities with extant taxa [15–17].

It is generally accepted that the chloroplast originated from endosymbiosis of ancient cyanobacteria [18]. The consensus chloroplast genome is circular and consists of two inverted repeats (IRa and IRb), a large single-copy region (LSC), and a small single-copy region (SSC). It is estimated that on average there are 400 to 1,600 copies of the chloroplast genome in each cell [19]. The chloroplast genome is generally uniparentally inherited, typically paternally in conifers and maternally in angiosperms [20,21] but some variation in the mode chloroplast inheritance has been reported in conifers [22].

In recent years several cupressophyte chloroplast genomes including those of *Agathis dammara* (Lamb.) Rich. & A.Richard, *Podocarpus lambertii* Klotzsch ex Endl, *Podocarpus totara* G.Benn. ex D.Don and *Nageia nagi* (Thunb.) Kuntze have been published [23,24]. Comparative studies of these genomes have provided fresh insights into various aspects of conifer chloroplast genome evolution through the examination of genome size, structure, organisation and gene content [23–26].

In this study, we use a range of next generation sequencing methods to determine the complete chloroplast genome (plastome) of *W*. *nobilis*. We compare the plastome of *W*. *nobilis* with available chloroplast genomes of Araucariaceae and Podocarpaceae, and analyse genome structure and organisation to infer the steps in genome evolution. We identify repetitive sequences in *W*. *nobilis* chloroplast genes and compare these with other repetitive sequences in Araucariaceae and Podocarpaceae. The availability of new genomic datasets will deliver new tools for exploring the genetic diversity of *W*. *nobilis*, and will support future conservation management strategies [27]. Genome-level sequencing is important in *W*. *nobilis* because a previous genetic study of approximately 800 AFLP, SSR and allozyme loci did not detect any genetic diversity [28], suggesting that living *W*. *nobilis* is extensively clonal or that genetic diversity could not be detected with these markers. The chloroplast genome reported in this study could be used to perform a more extensive search for genetic diversity in the Wollemi pine.

## Materials and Methods

### Chloroplast DNA extraction

Foliage from *Wollemia nobilis* provided by the Australian Botanic Gardens, Mt Annan (Sydney, NSW, Australia) was frozen at -80°C and stored until the time of DNA extraction. *W*. *nobilis* chloroplast DNA was isolated and amplified using the method described in [29].

### Genomic DNA extraction

Total DNA was extracted from young leaves using a modified cetyl trimethylammonium bromide (CTAB) method based on [30] and [28].

### Chloroplast DNA sequencing and genome assembly

Two next generation sequencing (NGS) data sets were used to assemble a draft *W*. *nobili*s chloroplast genome (S1 Table). These included total genomic DNA sequenced using the Illumina GAIIx platform, and chloroplast DNA sequenced using the Roche 454 GS-FLX. To confirm the draft genome, whole genomic DNA was then sequenced in a Nextera library on the Illumina MiSeq platform. All three NGS libraries were sequenced at the Ramaciotti Centre for Genomics (University of New South Wales).

Initial Illumina and 454 reads were trimmed using clean_reads v0.2.1 [31]. Illumina sequencing data were assembled using the Velvet short read assembler (v1.1.04) [32], and the 454 chloroplast data were assembled using Mira v3.2.1 [33]. These datasets were combined using Minimus2 v3.0.1 to produce contigs with sizes greater than 10kb [34]. Scaffold confirmation, arrangement and concatenation were implemented in Burrows Wheeler Aligner (BWA) [35]. This left a single gap in the resulting chloroplast sequence.

In order to resolve this gap, the chloroplast genomes of *W*. *nobilis* and *Agathis dammara* (AB830884) were aligned using MAUVE 2.3.1 software [36]. We observed a 5,000 bp sequence consisting of a section of a protein-coding gene (*ycf1*) that was absent in the *W*. *nobilis* genome. One hundred base pairs flanking this region were extracted from *A*. *dammara* and reads from the *W*. *nobilis* Illumina MiSeq library were mapped onto this sequence. This produced continuous mapping and high coverage over the gap region.

The *W*. *nobilis* chloroplast genome was then validated by mapping MiSeq reads to the final chloroplast genome. The Illumina MiSeq library was imported into CLC bio Genomics Workbench (v6.5, www.clcbio.com) using quality score settings for the Illumina Pipeline 1.8 and later. Sequences were trimmed based on a quality threshold of 0.05. Reads shorter than 150 bp and low quality reads were discarded. For the mapping, 90% of the read length was required to map with 80% similarity. A reliable reference sequence was produced since the mapping was continuous and there was consistently high coverage (average 408.54X; see S1 Table).

Raw sequence reads from the Illumina MiSeq library (total DNA) and the 454 sequencing (chloroplast DNA) have been deposited in the Sequence Read Archive (SRA) database with accession numbers SRR1927951 and SRR192612 respectively.

### Genome annotation

Initial annotation of the *Wollemia nobilis* chloroplast genome was performed using Glimmer3 (Gene Locator and Interpolated Markov ModelER) v3.02 and Dual Organellar GenoMe Annotator (DOGMA) [37]. Genes and open reading frames (ORF) that may not have been annotated were identified with the aid of blastx (http://blast.ncbi.nlm.nih.gov/Blast.cgi).

Putative starts, stops, and intron positions were determined by comparison with homologous genes in other chloroplast genomes using MAFFT online software [38]. In addition, all tRNA genes were further verified online using tRNAscan-SE search server [39] (http://lowelab.ucsc.edu/tRNAscan-SE/). The circular *W*. *nobilis* chloroplast genome map was drawn using OGDraw v1.2 [40].

### Sequence analyses and computational methods

Sequences homologous to the *W*. *nobilis* chloroplast genome were identified using Standard Nucleotide BLAST (http://blast.ncbi.nlm.nih.gov/). Whole genomes were aligned using progressive MAUVE implemented by MAUVE v2.3.1 software [36]. The AT content for the genome was calculated with Sequence Statistics on CLC Genomics Workbench v7.5 software (CLC bio). Genome annotation was performed in Geneious Pro v6.1.6 (Biomattters Ltd.), and the AT-content of protein-coding genes, tRNA genes, introns and intergenic spacers (IGSs) was determined on the basis of their annotation.

Simple sequence repeats (SSRs) were identified using Phobos Tandem Repeats Finder v3.3.12 [41]. The perfect search default settings were used and this involved a repeat unit size that ranged from one to 10 without setting a minimum satellite length constraint. A GFF file format was selected as the output option and cells were sorted based on the repeat number (with anything below three removed). Tandem repeats were identified with Tandem Repeats Finder (TRF) with default parameter settings [42]. The tandem repeat lengths were 20 bp or more with the minimum alignment score and maximum period size set as 50 and 500 (respectively), and the identity of repeats was set to ≥ 90%. REPuter [43] was used to visualize duplicated sequences in *W*. *nobilis* by forward versus reverse complement (palindromic) alignment, with the repeat size set to 200 to 5,000 bp.

## Results and Discussion

### General features of the *W*. *nobilis* chloroplast genome

**Fig 1:**
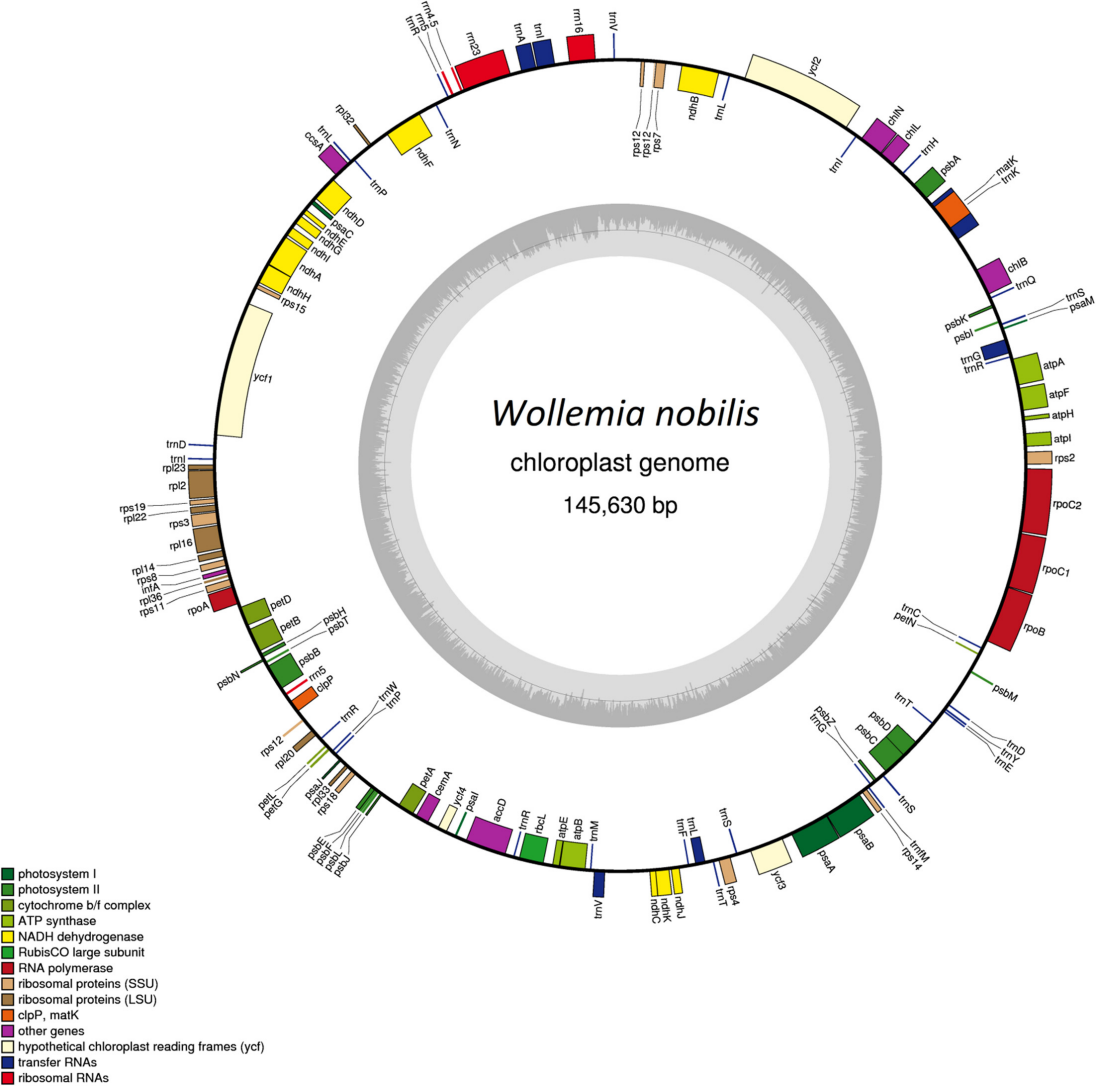
Sequence map of the *Wollemia nobilis* chloroplast genome. Genes drawn outside of the circle are transcribed clockwise, while genes shown on the inside of the circle are transcribed counter-clockwise. Genes belonging to different functional groups are colour-coded. The darker gray in the inner circle indicates GC content, while the lighter gray corresponds to AT content.

The complete circular chloroplast genome of *Wollemia nobilis* (GenBank accession KP259800) is 145,630 bp. The annotated genome is shown in Fig 1 and the sequencing results are detailed in S1 Table. The genome is very similar to that of *Agathis dammara* (145,625 bp) and is larger than the chloroplast genomes of *Podocarpus lambertii*, *Podocarpus totara* and *Nageia nagi* (Podocarpaceae). However, the genome is smaller than the largest known gymnosperm chloroplast genome from *Cycas taitungensis* C.F.Shen, K.D.Hill, C.H.Tsou & C.J.Chen (163,403 bp; NC_009618) [44].

The *W*. *nobilis* chloroplast genome encodes 122 genes, including 82 protein-coding genes, five ribosomal RNA genes, and 35 transfer RNA genes (Fig 1, Table 1 and Table 2). The 82 intact chloroplast protein-coding sequences are shared and are of similar length in Araucariaceae and Podocarpaceae, indicating evolutionarily conserved chloroplast gene content. A similar gene number is also shared with other cupressophytes including Cupressaceae and Cephalotaxaceae. In the gymnosperm families Cycadaceae, Ephedraceae and Ginkgoaceae, protein-coding genes are duplicated in the inverted repeat regions (IR). This increases the size of these genomes [24] but this feature is not present in the *Wollemia* chloroplast genome.

**Table 1:** Comparison of chloroplast genome characteristics in different species of Araucariaceae and Podocarpaceae.

	Araucariaceae		Podocarpaceae		
Characteristics	*Wollemia nobilis*	*Agathis dammara* ^*A*^	*Nageia nagi* ^*A*^	*Podocarpus lambertii* ^*B*^	*Podocarpus totara*
GenBank Accession no.	KP259800	AB830884	AB830885	NC_020361.1	NC_020361.1
Size (bp)	145,630	145,625	133,722	133,734	133,259
GC content (%)	36.50	36.54	37.26	37.100	37.16
Total number of genes	122	122 (123) ^*C*^	117 (120) ^*C*^	119 (118) ^*C*^	94 (120) ^*C*^
Total number of unique genes	118	(119) ^*C*^	(118) ^*C*^	118 (117) ^*C*^	(118)
Protein-coding genes	82	81 (82) ^*C*^	81 (82) ^*C*^	82	75 (82) ^*C*^
Ribosomal RNAs	5	5	4	4	4
Transfer RNAs	35	36	32 (34) ^*C*^	31 (32) ^*C*^	15 (34) ^*C*^
Protein-coding genes (bp)	75,300	75,271	74,781	74,217	74,607
Ribosomal RNAs (bp)	4,636	4,638	4,529	4,504	4,501
Transfer RNAs (bp)	2,628	2,699	2,418	2,409	2,487
Introns (bp)	11,857	11,890	11,487	10,445	9,710
Spacers (bp)	51,189	51,127	40,507	42,159	41,821
AT content (%)					
Genome	63.51	63.46	62.74	62.90	62.84
Protein-coding genes	62.41	62.34	61.86	61.87	61.90
Transfer RNA genes	46.47	46.46	46.80	46.66	47.30
Ribosomal RNA genes	45.75	45.90	46.01	45.87	45.90
Introns	62.39	62.61	61.92	62.13	61.50
Spacers	67.88	67.85	67.44	67.62	62.84

^*A*^ [25]

^*B*^ [23]

^*C*^ In parentheses is the value observed after these published chloroplast genomes were re-annotated using tRNAscan-SE [39] and reference to the *Cedrus deodara* (NC_014575) plastome.

**Table 2:** List of genes identified in the chloroplast genome of W. nobilis.

Functional category	Group of genes	Name of genes					
Self-replication	Ribosomal RNA genes	*rrn16*	*rrn23*	*rrn4*.*5*	rrn5**		
	Transfer RNA genes	trnA-UGC*	*trnC-GCA*	trnD-GUC**	*trnE-UUC*	*trnF- GAA*	*trnfM- CAU*
		*trnG- GCC*	trnG- UCC*	*trnH- GUG*	trn-I CAU**	trnI- GAU*	trnK- UUU*
		*trnL- CAA*	trnL- UAA*	*trnL- UAG*	trnM- CAU*	*trnN- GUU*	*trnP- GGG*
		*trnP- UGG*	*trnQ- UUG*	*trnR- ACG*	*trnR- CCG*	trnR-UCU**	*trnS- UGA*
		*trnS-GCU*	*trnS-GGA*	*trnT- GGU*	*trnT- UGU*	*trnV- GAC*	trnV- UAC*
		*trnW- CCA*	*trnY-GUA*				
	Small subunit of ribosome	*rps11*	rps12*	*rps14*	*rps15*	*rps18*	*rps19*
		rps2*	*rps3*	*rps4*	*rps7*	*rps8*	
	Large subunit of ribosome	*rpl14*	rpl16*	*rpl2*	*rpl20*	*rpl22*	*rpl23*
		*rpl32*	*rpl33*	*rpl36*			
	DNA-dependent RNA polymerase	*rpoA*	*rpoB*	rpoC1*	*rpoC2*		
	Translational initiation factor	*infA*					
Genes for photosynthesis	Subunits of photosystem I	*psaA*	*psaB*	*psaC*	*psaI*	*psaJ*	*psaM*
		ycf3*	*ycf4*				
	Subunits of photosystem II	*psbA*	*psbB*	*psbC*	*psbD*	*psbE*	*psbF*
		*psbH*	*psbI*	*psbJ*	*psbK*	*psbL*	*psbM*
		*psbN*	*psbT*	*psbZ*			
	Subunits of cytochrome	*petA*	petB*	petD*	*petG*	*petL*	*petN*
	Subunits of ATP synthase	*atpA*	*atpB*	*atpE*	atpF*	*atpH*	*atpI*
	Large subunit of Rubisco	*rbcL*					
	Chlorophyll biosynthesis	*chlB*	*chlL*	*chlN*			
	Subunits of NADH dehydrogenase	ndhA*	ndhB*	*ndhC*	*ndhD*	*ndhE*	*ndhF*
		*ndhG*	*ndhH*	*ndhI*	*ndhJ*	*ndhK*	
Other genes	Maturase	*matK*					
	Envelope membrane protein	*cemA*					
	Subunit of acetyl-CoA	*accD*					
	C-type cytochrome synthesis gene	*ccsA*					
	Protease	*clpP*					
	Component of TIC complex	*ycf1*					
Genes of unknown function	Conserved open reading frames	*ycf2*					

*genes with introns

**duplicated genes

Table 1 details the results of a comparative analysis of the *W*. *nobilis*, *A*. *dammara*, *P*. *lambertii*, *P*. *totara* and *N*. *nagi* chloroplast genomes. The gene content of these genomes was determined using both the annotation methods described in this study, and by reference to the previously published annotations on NCBI Genome (http://www.ncbi.nlm.nih.gov/genome). Differences between these annotations (probably due to differences in annotation methodology) have been noted in Table 1, with the values observed in our annotation shown in parentheses. The major differences between our annotations and the previously published annotations were: (1) the published annotation for *P*. *totara* (NC_020361.1) was very incomplete and had many missing protein-coding genes and tRNAs; (2) the *matK* gene was absent in both *A*. *dammara* and *N*. *nagi* even though high similarity was observed when aligned to a similar sequence in *W*. *nobilis*; (3) the two tRNAs (*trnC* and *trnQ*) were not annotated in the previously published *N*. *nagi* annotation; and (4) the gene number in the published *P*. *lambertii* annotation included a pseudogene, but we have omitted this from the total number of genes shown for the *P*. *lambertii* plastome in Table 1.

### Base composition

GC base pairs are more thermodynamically stable than AT base pairs, and so GC content influences chloroplast genome stability. The GC content of the *Wollemia* chloroplast genome (36.5%) is very similar to *A*. *dammara* but slightly lower than the GC content of Podocarpaceae chloroplast genomes (which range from 37.1 to 37.26%; Table 1). The GC content of the *W*. *nobilis* chloroplast genome is also higher than members of Cupressaceae such as *Taiwania cryptomerioides* Hayata (34.63%), *Calocedrus formosana* (Florin) W.C.Cheng & L.K.Fu (35.38%) and *Cryptomeria japonica* (Thunberg ex Linnaeus f.) D.Don (34.83%) [25].

Previous studies have found that the AT content in genomic regions may be associated with the dynamics of repeats (e.g. [45,46]) and may also be associated with the codon bias of chloroplast protein-coding genes and hence the regulation of gene expression (e.g. [45,46]). AT-rich regions in the *Wollemia* chloroplast genome include intergenic (67.88%), protein-coding (62.41%) and intronic (62.39%) regions, while rRNAs (45.75%) and tRNAs (46.47%) have a much lower AT content. These patterns are similar across all species listed in Table 1, as well as in the plastomes of many other plants (e.g. [25,47]).

### Structure of *rps16*

Ribosomal protein S16 (*Rps16*) is essential for the translation of chloroplast genes in tobacco [48] and can be found in some cupressophytes (e.g. *Cephalotaxus oliveri* Mast. and *Cryptomeria japonica*; [26,49]). The *rps16* gene is situated between the *chlB* gene and the *trnK-UUU* gene in a conserved part of the genome. The coding sequence is 268 bp in length and has an 853 bp intron in *C*. *oliveri*. When the *chlB/trnK* region of *Wollemia nobilis* is aligned with that of *C*. *oliveri*, only remnants of the *rps16* gene are evident due to the absence of an initiation codon. A similar *rps16* remnant region is also present in *Agathis dammara* and has ~95% similarity to the corresponding *W*. *nobilis* sequence. In comparison, the *chlB/trnK* intergenic regions in *P*. *lambertii*, *P*. *totara* and *N*. *nagi* do not resemble the *rps16* gene at all. A possible slower mutation rate in Araucariaceae compared to Podocarpaceae could explain the complete absence of *rps16* in the latter group. The absence of a functional *rps16* gene in this location could indicate that this gene is not essential for translation in *Agathis* and *Wollemia* chloroplasts. Alternatively, its function could be replaced by another ribosomal protein or by an intact nuclear copy of *rps16*, as in some legumes [50].

Within the Cupressaceae the *rps16* gene is present in *Calocedrus formosana* and *C*. *japonica*, but is absent from *Juniperus scopulorum* Sarg. Similarly in the Taxaceae it is present in *Taxus mairei* (Lemée & Lév.) S.Y.Hu ex T.S.Liu but absent in *Amentotaxus formosana* H.L.Li. This suggests that there have been multiple independent losses of the *rps16* gene within the gymnosperms. Further study to trace *rps16* gene loss through the conifer lineages could aid in understanding the process of chloroplast genome evolution in gymnosperms.

### Comparative analysis of introns and intergenic regions

There are 16 intron-containing chloroplast genes in *W*. *nobilis*, including six tRNA genes and 10 protein-coding genes. Similar intronic features were observed in *A*. *dammara*. Nearly all of these genes contain a single intron except for the two introns in *ycf3* and *rps12*. The *trnK-UUU* gene has an unusual intron that encodes a *matK* ORF. This *trnK* intron is observed in many plants and has been extensively used as a phylogenetic marker (e.g. [51,52]). Additionally, we observed a 31 bp overlap between *ndhC* and *ndhK*, and a 53 bp overlap between *psbC* and *psbD*.

*W*. *nobilis* and *A*. *dammara* have 120 highly similar intergenic regions. However, the intergenic region between *rbcL* and *trnR-CCG* in *W*. *nobilis* contains a *trnD-GUC* in *A*. *dammara*, producing the intergenic regions *rbcL/trnD and trnD/trnR*. Four intergenic regions (*rpoC1/rpoC2*, *psaB/psaA*, *psbF/psbE* and *ndhH/ndhA*) are identical in sequence between these two species.

The *psbA*/*trnH* intergenic region is the most widely used plastid barcode for species differentiation in land plants including *Araucaria* [53,54]. It is highly variable in sequence and in length [27,54,55], with a non-coding region flanked by two conserved coding regions, *psbA* (which encodes photosystem II protein D1) and *trnH*-*GUG*. We observed 646 bp of additional sequence in the 847 bp *psbA*/*trnH* intergenic region in *W*. *nobilis*. This sequence was absent in *A*. *dammara* where the *psbA*/*trnH* intergenic region was 201 bp in length. BLAST analyses of the *W*. *nobilis psbA/trnH* intergenic region indicate that this indel is present in all 19 *Araucaria* species. The length of *psbA/trnH* in Podocarpaceae ranges from 600 to 626 bp. This suggests that a deletion may have occurred in this region in *Agathis* after the divergence of *Agathis* and *Wollemia*.

### Comparative analysis of tRNAs

*Wollemia nobilis* and *A*. *dammara* have the same 32 unique tRNAs, but have different numbers of tRNA coding sequences due to gene duplication events (Table 1). *W*. *nobilis* has 35 tRNAs because it only has two of the three copies of *trnD-GUC* observed in *A*. *dammara*. The *trnD-GUC* gene in *W*. *nobilis* is associated with a 760 bp direct repeat, and the *trnR-UCU* is also duplicated and is associated with a direct repeat of 310 bp. These tRNA-containing repeats are not present in *A*. *dammara* and the impact of these repeats on chloroplast genome function is unclear.

Analyses of plastid tRNAs could support a better understanding of the divergence among conifers [23]. The *trnR-CCG* gene is entirely absent in Cupressaceae, Taxaceae and Cephalotaxaceae, but is found in both Pinaceae and Podocarpaceae. It is present in *W*. *nobilis* and *Agathis*, and may be generally present in Araucariaceae. This provides further evidence for a major loss of the *trnR-CCG* gene in the Taxaceae/Taxodiaceae/Cupressaceae group [23]. The *trnR-CCG* gene may have been readily lost because it is not essential for translation in land plants [56].

### Remnant inverted repeats in *W*. *nobilis* and Araucariaceae

A large inverted repeat (IR) is found in many land plants and typically includes a pair of *ycf2* and ribosomal operons. However, in several gymnosperms (including Pinaceae, Cupressaceae, Cephalotaxaceae and Podocarpaceae) only short remnants of the IR have been observed [24,26]. We identified two short IRs in *W*. *nobilis*: a 602 bp IR region that includes the *rrn5* gene, and another region of 73 bp that includes the *trnI-CAU* gene. Both of these IRs are also found in *A*. *dammara*. The *rrn5*-containing short IR was not found in any of the cupressophytes (Cupressaceae, Cephalotaxaceae, Podocarpaceae or Taxaceae).

Duplicated and inverted tRNAs were observed in *W*. *nobilis* as well as in *A*. *dammara*. The duplicated tRNA, *trnI-CAU*, is inverted in *W*. *nobilis* as well as in *Taiwania cryptomerioides* and *Pinus thunbergii* Parlatore [49,57]. Other tRNAs including *trnN-GUU* in Podocarpaceae [23,25] and *trnQ-UUG* in Cephalotaxaceae [49] have also been identified.

### Whole genome comparative analysis

**Fig 2:**
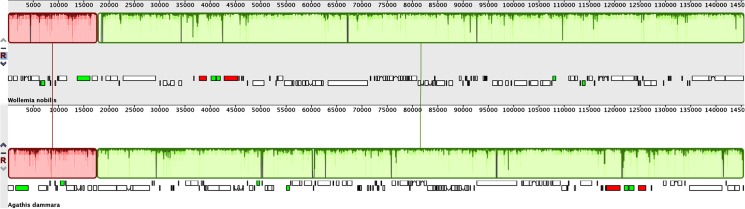
MAUVE alignment of *W*. *nobilis* and *A*. *dammara* chloroplast genomes. The *W*. *nobilis* genome is shown at top as the reference genome. Within each of the alignments, local collinear blocks are represented by blocks of the same colour connected by lines. Note that the two LCBs in the *A*. *dammara* genome are both inverted relative to the *W*. *nobilis* genome.

The *W*. *nobilis* chloroplast genome was aligned with the chloroplast genomes of other closely related gymnosperms to compare the organisation of these genomes. Fig 2 shows two locally collinear blocks (LCBs) between the *W*. *nobilis and A*. *dammara* chloroplast genomes. These blocks suggest a high level of similarity in genome organisation between these two species, although they are inverted relative to each other (Fig 2). More comparisons of *W*. *nobilis* to members of the Podocarpaceae produced chloroplast genome alignments with several inversions and translocations. There are seven LCBs between *W*. *nobilis* vs. *P*. *lambertii*, nine LCBs between *W*. *nobilis* vs. *N*. *nagi* and 10 LCBs between *W*. *nobilis* vs. *P*. *totara* (S1 Fig). These comparisons show that the chloroplast genomes of *P*. *lambertii* and *P*. *totara* are both very different in structure as previously reported [23]. Examination of local pairwise alignments between the chloroplast genomes of *W*. *nobilis* and *A*. *dammara* also shows a high level of sequence similarity (96.6%). Collectively these alignments confirm the close evolutionary relationship between *Wollemia* and *Agathis* species [14,58,59], and the more distant relationship between *Wollemia* and *Podocarpus* or *Nageia*.

### Repetitive sequences in the chloroplast genome of *W*. *nobilis*

Although large numbers of tandem repeats have been reported in conifers [23,49], the mechanisms underlying the origin of these tandem repeats remain unclear. Nonetheless, they are known to be associated with gene duplication [60], gene expansion [23,49] and chloroplast DNA rearrangement [61]. We identified 28 tandem repeats of more than 20 bp in length in the *W*. *nobilis* chloroplast genome (Table 3), of which 12 are in intergenic regions, 14 in coding regions, and two extend from an intergenic region into a coding region. The length of the repeat units in these regions varied between 11 and 60 bp, and up to 11 repeat units were present.

**Table 3:** Distribution of tandem repeats in the *W*. *nobilis* chloroplast genome.

Serial No.	Indices	Repeat length	Size of repeat unit X Copy number	Location
1	4445–4492	54	18x3	*atpF*/*atpA* (IGS)
2	18715–18751	36	12x3	*trnH*/*chlL* (IGS)
3	25945–25971	24	12x2	*ycf2* (CDS)
4	26323–26359	36	18x2	*ycf2* (CDS)
5	34174–34345	171	57x3	*rps7* (CDS)
6	36587–36660	72	36x2	*rps12*/*trnV* (IGS)
7	37477–37502	24	12x2	*trnV*/*rrn16* (IGS)
8	37734–37762	28	14x2	*trnV*/*rrn16* (IGS)
9	47091–47126	32	16x2	*trnR*/*trnN* (IGS)
10	64129–64185	60	15x4	*ycf1* (CDS)
11	65803–65865	66	33x2	*ycf1* (CDS)
12	65853–65894	45	15x3	*ycf1* (CDS)
13	66632–66672	42	21x2	*ycf1* (CDS)
14	67290–67332	42	21x2	*ycf1* (CDS)
15	69017–69356	330	30x11	*ycf1* (CDS)
16	72719–72756	38	19x2	*rpl23* (CDS)
17	74646–74676	32	16x2	*rpl2*/*rps19* (IGS)
18	84689–84713	26	13x2	*psbT*/*psbB* (IGS)
19	91442–91485	44	22x2	*trnP/psaJ* (IGS)
20	92695–92802	108	54x2	*rps18* (CDS) and *rps18*/*psbF* (IGS)
21	100704–101063	360	60x6	*accD* (CDS)
22	101250–101295	45	45	*accD* (CDS)
23	101313–101337	24	12x2	*accD* (CDS)
24	113049–113073	24	12x2	*ndhJ*/*trnF* (IGS)
25	115787–115820	32	16x2	*rps4/trnS* (IGS)
26	123920–124039	120	60x2	*psaB*/*rps14* (IGS) and *rps14* (CDS)
27	125626–125659	39	13x3	*trnS*/*psbC* (IGS)
28	140203–140242	42	21x2	*rpoC1* (CDS)

The *accD* gene encodes the acetyl-CoA carboxylase beta subunit. The ORF of this gene is variable among land plants, with cupressophytes having the largest expansions of the *accD* ORF (ranging from 700 to 1,056 codons; [49]). The large size of the *accD* ORF in cupressophytes has been attributed to the accumulation of tandem repeat sequences within the gene [26,49]. The *accD* reading frame of *W*. *nobilis* is 800 codons in length, and hence is shorter than that of *A*. *dammara* (820 codons) but longer than that of *P*. *lambertii* (684 codons). Large insertions are usually found in the middle of the *accD* ORF [49], and this was also the case for *W*. *nobilis* in which several tandem repeats were observed in *accD*. The longest tandem repeat was 360 bp in length (as shown in Table 3), and consisted of six copies of 20 imperfect amino acid sequences starting with an LDREEK motif. The other tandem repeats are located downstream of this repeat, such that there are three copies of the motif, PEEEV and then two copies of the motif QWVN. Nine similar repeats were found in the *accD* gene in *C*. *oliveri*, and this gene remained functional [49]. Hence, the *Wollemia accD* gene is also expected to retain its normal function.

The protein-coding region *ycf1* contains higher numbers of tandem repeats and SSRs than any other gene within the *W*. *nobilis* chloroplast genome. This includes 17 poly-A repeats, and six different tandem repeats (Tables 3 and 4). The *ycf1* gene is often the largest protein-coding gene in plastomes (e.g. 7,830 bp in *W*. *nobilis*, 7,914 bp in *A*. *dammara*) and encodes a chloroplast envelope protein translocase (part of the TIC complex; [62]). High numbers of tandem repeats and SSRs (11 tandem repeats and 148 SSRs) were also reported in the *ycf1* gene in *P*. *lambertii* [23]. Internal stop codons are absent in both *W*. *nobilis* and *P*. *lambertii ycf1*, suggesting that the *ycf1* gene in these species encodes a functional protein.

**Table 4:** Characteristics of simple sequence repeats identified in the chloroplast genomes of *W*. *nobilis*, *A*. *dammara* and *P*. *lambertii*.

	Mono	Di	Tri	Tetra	Penta	Hexa	Total
*W*. *nobilis*							
Total counts	239	69	62	15	1	1	387
Total Repeat Length (repeat unit X number of repeat) (bp)	1991	744	621	184	15	18	3573
Density (Total repeat length/genome size) [bp/kb]	13.67	5.11	4.26	1.26	0.10	0.12	24.53
Proportion among other SSR (%)	55.72	20.82	17.38	5.15	0.42	0.50	100
Mean Length	8.33	10.78	10.02	12.27	15	18	9.23
Standard Deviation	1.84	4.45	3.64	1.03	0	0	
*A*. *dammara*							
Total counts	250	68	64	12	2	1	397
Total Repeat Length (repeat unit X number of repeat) (bp)	2168	720	615	152	30	18	3703
Density (Total repeat length/genome size) [bp/kb]	14.89	4.94	4.22	1.04	0.21	0.12	25.43
Proportion among other SSR (%)	58.55	19.44	16.61	4.10	0.81	0.49	100
Mean Length	8.67	10.59	9.61	12.67	15	18	9.33
Standard Deviation	2.38	3.88	1.53	1.56	0	0	
*P*. *lambertii*							
Total counts	198	63	52	9	1	1	324
Total Repeat Length (repeat unit X number of repeat) (bp)	1558	586	498	112	15	18	2787
Density (Total repeat length/genome size) [bp/kb]	11.65	4.38	3.72	0.84	0.11	0.13	20.84
Proportion among other SSR (%)	55.90	21.03	17.87	4.02	0.54	0.65	100
Mean Length	7.87	9.30	9.58	12.44	15	18	8.60
Standard Deviation	1.56	2.81	1.33	1.33	0	0	

### Short simple repeats in the *W*. *nobilis* chloroplast genome

Simple sequence repeats (SSRs) usually have a higher rate of mutation compared with other neutral regions of DNA due to slipped strand mispairing. Chloroplast SSRs are often used as molecular markers in genetic studies analysing population structure as these short repeats are haploid and uniparentally inherited [63,64]. Here, we compared the perfect SSRs between the three species *W*. *nobilis*, *A*. *dammara* and *P*. *lambertii* (Table 4) using summarised data collected from Phobos (S2 Table). The largest number of SSRs was found in *A*. *dammara*, followed by *W*. *nobilis* and *P*. *lambertii*. Given the varying genome sizes, we observed the overall SSR density and found *W*. *nobilis* (24.53 bp every 1000 bp) and *A*. *dammara* (25.43 bp every 1000 bp) were more similar to each other than to *P*. *lambertii* (20.84 bp every 1000 bp). The average repeat lengths of the mono-, di- and tri- nucleotides for *W*. *nobilis* (9.71 bp) and *A*. *dammara* (9.62 bp) were similar whereas in *P*. *lambertii* the average repeat length was 8.91 bp. Mononucleotide repeats were found to be the most common type of SSR in all three species (Table 4), and poly-A repeats were more abundant than poly-C repeats (S2 Table). The function of these repeats (if any) could be investigated by further characterisation of SSRs at specific genomic regions such as coding sequences, introns or intergenic spacers. The SSRs in *W*. *nobilis* could also be used to investigate its genetic diversity.

It is important to note that previous studies have used varied algorithms for SSR detection [64,65]. Hence, any further comparisons between *W*. *nobilis* and other species would have to be made using the SSR criteria described in this study or another common set of SSR criteria.

## Conclusion

We used a combination of *de novo* assembly and reference to the *A*. *dammara* chloroplast genome to obtain the complete chloroplast genome sequence for *Wollemia nobilis*, a critically endangered Southern conifer with a very small extant population. Although *Wollemia* is a monotypic genus, we observe a close similarity between the chloroplast genomes of *A*. *dammara* and *W*. *nobilis* in terms of genome size, organisation and sequence. The shared genomic features include *rrn5* and *trnI* IR remnants, a syntenic *rps16* pseudogene and an insertion/deletion hotspot in the *psbA/trnH* intergenic region. Our data provide an insight into the evolution of the Araucariaceae plastid genome in the wider context of plastid evolution in conifers. A striking feature of the *W*. *nobilis* chloroplast genome is its large number of repetitive sequences, notably within the *accD* gene and including a large number of SSRs. These sequences could be used as molecular markers in future studies aimed at identifying and conserving genetic diversity in the Wollemi pine.

## Supporting Information

S1 Fig MAUVE alignments of *W. nobilis* chloroplast genome and other gymnosperm chloroplast genomes. a. *W. nobilis vs. P. lambertii*, b. *W. nobilis vs. P. totara*, c. *W.nobilis vs. N. nagi*.
(DOCX)

S1 Table Next generation sequencing datasets used for the assembly of the *W*. *nobilis* chloroplast genome. (DOCX)

S2 Table List of SSRs in *W*. *nobilis*, *P*. *lambertii* and *A*. *dammara* generated from Phobos v.3.3.12. (DOCX)
