# Supplementary material for: Complete Chloroplast Genome of the Wollemi Pine (Wollemia nobilis): Structure and Evolution
Source: PLoS One. 2015 Jun 10;10(6):e0128126. doi: 10.1371/journal.pone.0128126 (PMC4464890; doi:10.1371/journal.pone.0128126)
Supplement: S1 Fig — a. W. nobilis vs. P. lambertii, b. W. nobilis vs. P. totara, c. W.nobilis vs. N. nagi. (DOCX) [file pone.0128126.s001.docx]

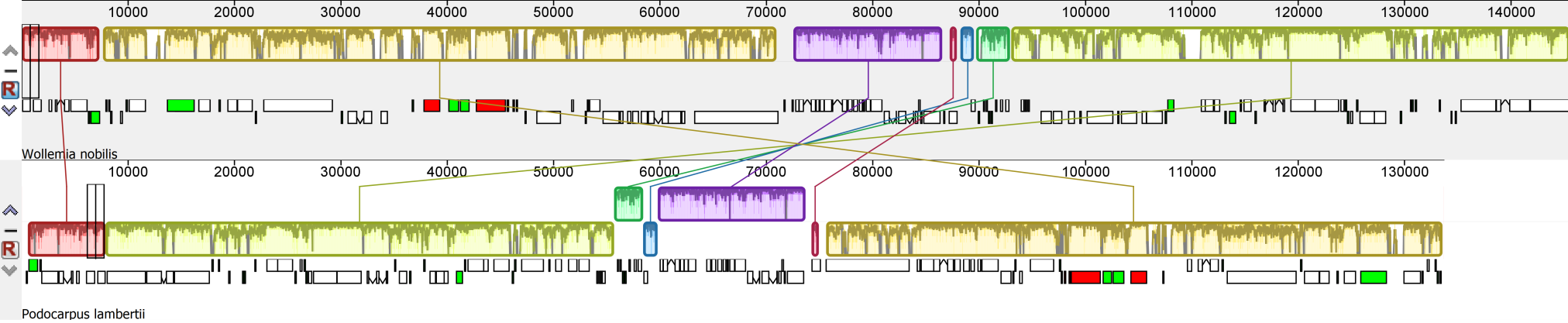


**Supplementary Data**

a.

b.

c.


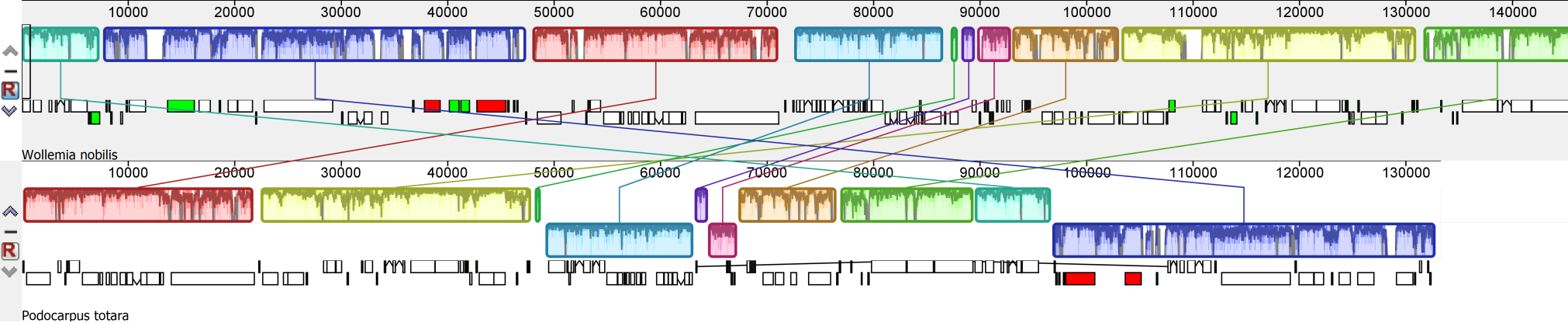


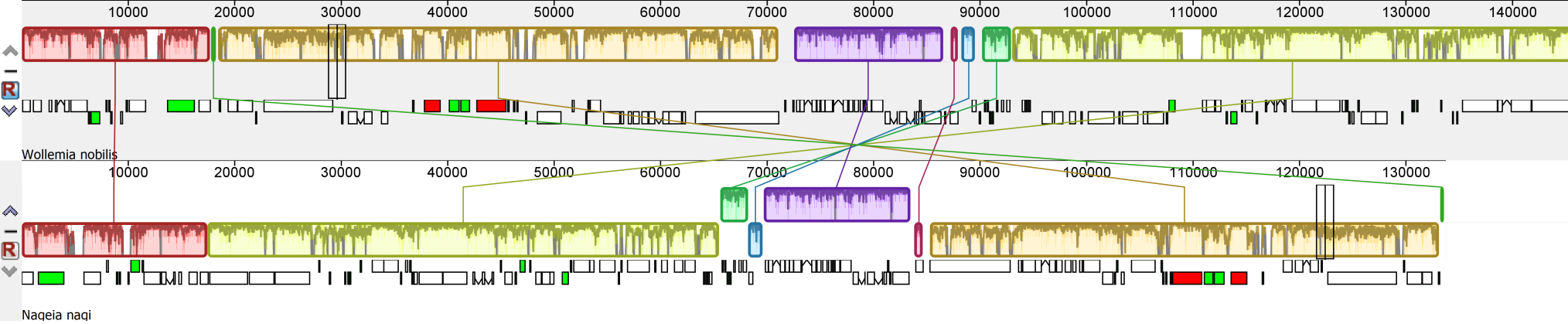


Supplementary Figure 1: MAUVE alignment for a. *W. nobilis vs. P. lambertii*, b. *W. nobilis* *vs P. totara,* c. *W.nobilis vs N****.*** *nagi*.
