# Supplementary material for: Complete Chloroplast Genome of the Wollemi Pine (Wollemia nobilis): Structure and Evolution
Source: PLoS One. 2015 Jun 10;10(6):e0128126. doi: 10.1371/journal.pone.0128126 (PMC4464890; doi:10.1371/journal.pone.0128126)
Supplement: S1 Table — (DOCX) [file pone.0128126.s002.docx]

## Supplementary Data

### Genome Assembly

**Supplementary Table 1:** The three NGS shotgun libraries used for the assembly of the *W. nobilis* chloroplast genome

|  | **Total DNA** | | **Chloroplast DNA** |
| --- | --- | --- | --- |
|  | Illumina GAIIx | Illumina MiSeq library ^A^ | 454 GS-FLX |
| **No. of raw reads** | 61,610,544 | 7,657,422 | 239,242 |
| **Ave. length before trimming** | 99.15 | 252.60 | 321.10 |
| **No. of reads after trimming** | 59,948,550 | 6,814,929 | 229,537 ^B^ |
| **No. of chloroplast reads** | 2,256,490 | 232,818 ^B^ | 30,164 |
| **% Total reads** | 3.76 | 3.49 | 13.14 |
| **Ave. chloroplast**  **read coverage** | 1589 | 408.54 | 58.13 |
| **Max. chloroplast read coverage** | 3460 | 709 | 403 |
| **Min. read coverage of complete chloroplast genome** | 0 ^C^ | 42 | 0 ^C^ |

^A^ used to confirm the complete genome after assembly

^B^ reads submitted to SRA database (accession numbers for total DNA and chloroplast DNA are SRR1927951 and SRR192612 respectively)

^C^ zero coverage initially as these data sets did not cover the gaps covered by the MiSeq library
