# Supplementary material for: Complete Chloroplast Genome of the Wollemi Pine (Wollemia nobilis): Structure and Evolution
Source: PLoS One. 2015 Jun 10;10(6):e0128126. doi: 10.1371/journal.pone.0128126 (PMC4464890; doi:10.1371/journal.pone.0128126)
Supplement: S2 Table — (DOCX) [file pone.0128126.s003.docx]

**Supplementary Table 2: List of SSRs in *W. nobilis,* *P. lambertii* and *A. dammara* generated from Phobos v.3.3.12**

| *W. nobilis* | Total Repeat Length (repeat unit X number of repeat) | | | | | | | | | | | | | | |  |
| --- | --- | --- | --- | --- | --- | --- | --- | --- | --- | --- | --- | --- | --- | --- | --- | --- |
| SSR sequence | **3** | **4** | **5** | **6** | **7** | **8** | **9** | **10** | **11** | **12** | **13** | **14** | **15** | **16** | **17** | **Total** |
| A/T | - | - | - | - | 97 | 53 | 32 | 13 | 11 | 6 | 3 | 3 | 1 | 2 | 1 | 222 |
| C/G | - | - | - | - | 8 | 5 | 4 | - | - | - | - | - | - | - | - | 17 |
| AG | - | 20 | 4 | - | 1 | 1 | - | - | - | - | - | - | - | - | - | 26 |
| AT | - | 20 | 4 | 2 | 4 | 2 | 3 | 4 | - | 1 | - | 1 | - | - | - | 41 |
| AC | - | 2 | - | - | - | - | - | - | - | - | - | - | - | - | - | 2 |
| AAC | 4 | - | - | - | - | - | - | - | - | - | - | - | - | - | - | 4 |
| AAG | 19 | 2 | 1 | - | - | - | - | - | - | 1 | - | - | - | - | - | 23 |
| AAT | 14 | 1 | 1 | - | - | - | - | - | - | - | - | - | - | - | - | 16 |
| ATC | 8 | 1 | - | - | - | - | - | - | - | - | - | - | - | - | - | 9 |
| ACT | 1 | - | - | - | - | - | - | - | - | - | - | - | - | - | - | 1 |
| AGC | 1 | - | - | - | - | - | - | - | - | - | - | - | - | - | - | 1 |
| ACG | 1 | - | - | - | - | - | - | - | - | - | - | - | - | - | - | 1 |
| AGG | 2 | 4 | - | - | - | - | - | - | - | - | - | - | - | - | - | 6 |
| ACC | 1 | - | - | - | - | - | - | - | - | - | - | - | - | - | - | 1 |
| AAAG | 3 | - | - | - | - | - | - | - | - | - | - | - | - | - | - | 3 |
| AAAT | 5 | 1 | - | - | - | - | - | - | - | - | - | - | - | - | - | 6 |
| AACC | 1 | - | - | - | - | - | - | - | - | - | - | - | - | - | - | 1 |
| AGAT | 3 | - | - | - | - | - | - | - | - | - | - | - | - | - | - | 3 |
| ACAT | 1 | - | - | - | - | - | - | - | - | - | - | - | - | - | - | 1 |
| ACCT | 1 | - | - | - | - | - | - | - | - | - | - | - | - | - | - | 1 |
| AAAAG | 1 | - | - | - | - | - | - | - | - | - | - | - | - | - | - | 1 |
| AGATCT | 1 | - | - | - | - | - | - | - | - | - | - | - | - | - | - | 1 |
|  |  |  |  |  |  |  |  |  |  |  |  |  |  |  |  | 387 |

| *A. dammara* | Repeat Unit Size | | | | | | | | | | | | | | | |  |
| --- | --- | --- | --- | --- | --- | --- | --- | --- | --- | --- | --- | --- | --- | --- | --- | --- | --- |
| SSR sequence | **3** | **4** | **5** | **6** | **7** | **8** | **9** | **10** | **11** | **12** | **13** | **14** | **15** | **16** | **17** | **18** | **Total** |
| A/T | - | - | - | - | 113 | 36 | 18 | 17 | 14 | 12 | 9 | 3 | 4 | 3 | 2 | 1 | 232 |
| C/G | - | - | - | - | 10 | 3 | 4 | 1 | - | - | - | - | - | - | - | - | 18 |
| AG | - | 19 | 4 | 1 | - | - | - | - | - | - | - | - | - | - | - | - | 24 |
| AT | - | 15 | 8 | 4 | 3 | 4 | 2 | 4 | 1 | - | - | - | - | - | - | - | 41 |
| AC | - | 3 | - | - | - | - | - | - | - | - | - | - | - | - | - | - | 3 |
| AAC | 4 | - | - | - | - | - | - | - | - | - | - | - | - | - | - | - | 4 |
| AAG | 18 | 5 | 2 | - | - | - | - | - | - | - | - | - | - | - | - | - | 25 |
| AAT | 17 | 1 | 1 | - | - | - | - | - | - | - | - | - | - | - | - | - | 19 |
| AGC | 1 | - | - | - | - | - | - | - | - | - | - | - | - | - | - | - | 1 |
| AGG | 2 | - | - | - | - | - | - | - | - | - | - | - | - | - | - | - | 2 |
| ATC | 9 | 1 | - | - | - | - | - | - | - | - | - | - | - | - | - | - | 10 |
| ACT | 1 | - | - | - | - | - | - | - | - | - | - | - | - | - | - | - | 1 |
| ACG | 1 | - | - | - | - | - | - | - | - | - | - | - | - | - | - | - | 1 |
| ACC | 1 | - | - | - | - | - | - | - | - | - | - | - | - | - | - | - | 1 |
| AAAG | 4 | - | - | - | - | - | - | - | - | - | - | - | - | - | - | - | 4 |
| AAAT | 3 | 2 | - | - | - | - | - | - | - | - | - | - | - | - | - | - | 5 |
| AACC | 1 | - | - | - | - | - | - | - | - | - | - | - | - | - | - | - | 1 |
| ACCT | 1 | - | - | - | - | - | - | - | - | - | - | - | - | - | - | - | 1 |
| AGAT | 1 | - | - | - | - | - | - | - | - | - | - | - | - | - | - | - | 1 |
| AAAAG | 1 | - | - | - | - | - | - | - | - | - | - | - | - | - | - | - | 1 |
| AAGAG | 1 | - | - | - | - | - | - | - | - | - | - | - | - | - | - | - | 1 |
| AGATCT | 1 | - | - | - | - | - | - | - | - | - | - | - | - | - | - | - | 1 |
|  |  |  |  |  |  |  |  |  |  |  |  |  |  |  |  |  | 397 |

| *P. lambertii* | Total Repeat Length (repeat unit X number of repeat) | | | | | | | | | | | | |  | |
| --- | --- | --- | --- | --- | --- | --- | --- | --- | --- | --- | --- | --- | --- | --- | --- |
| SSR sequence | **3** | **4** | **5** | **6** | **7** | **8** | **9** | **10** | **11** | **12** | **13** | **14** | **15** | | **Total** |
| A/T | - | - | - | - | 118 | 39 | 14 | 6 | 4 | 6 | - | - | 1 | | 188 |
| C/G | - | - | - | - | 3 | - | 3 | - | - | 1 | 1 | - | 2 | | 10 |
| AG | - | 21 | 1 | - | - | - | - | - | - | - | - | - | - | | 22 |
| AT | - | 24 | 7 | 2 | 2 |  | 3 | 1 | - | - | - | - | - | | 39 |
| AC | - | 1 | - | 1 | - | - | - | - | - | - | - | - | - | | 2 |
| AAC | 1 | - | - | - | - | - | - | - | - | - | - | - | - | | 1 |
| AAG | 11 | 3 | - | - | - | - | - | - | - | - | - | - | - | | 14 |
| AAT | 14 | 5 | 1 | - | - | - | - | - | - | - | - | - | - | | 20 |
| AGC | 4 | - | - | - | - | - | - | - | - | - | - | - | - | | 4 |
| AGG | 5 | - | - | - | - | - | - | - | - | - | - | - | - | | 5 |
| ATC | 5 | - | - | - | - | - | - | - | - | - | - | - | - | | 5 |
| ACT | 3 | - | - | - | - | - | - | - | - | - | - | - | - | | 3 |
| AATC | 2 | 1 | - | - | - | - | - | - | - | - | - | - | - | | 3 |
| AATG | 4 | - | - | - | - | - | - | - | - | - | - | - | - | | 4 |
| AATT | 1 | - | - | - | - | - | - | - | - | - | - | - | - | | 1 |
| ACCT | 1 | - | - | - | - | - | - | - | - | - | - | - | - | | 1 |
| AAATG | 1 | - | - | - | - | - | - | - | - | - | - | - | - | | 1 |
| AGATAT | 1 | - | - | - | - | - | - | - | - | - | - | - | - | | 1 |
|  |  |  |  |  |  |  |  |  |  |  |  |  |  | | 324 |
